# Phytochemical, Cytotoxic, and Antimicrobial Evaluation of *Tribulus terrestris* L*., Typha domingensis* Pers., and *Ricinus communis* L.: Scientific Evidences for Folkloric Uses

**DOI:** 10.1155/2022/6519712

**Published:** 2022-01-27

**Authors:** Asaad Khalid, Alanood S. Algarni, Husham E. Homeida, Shahnaz Sultana, Sadique A. Javed, Zia ur Rehman, Hana Abdalla, Hassan A. Alhazmi, Mohammed Albratty, Ashraf N. Abdalla

**Affiliations:** ^1^Substance Abuse and Toxicology Research Centre, Jazan University, P. O. Box 114, Jazan 45142, Saudi Arabia; ^2^Medicinal and Aromatic Plants and Traditional Medicine Research Institute, National Center for Research, P. O. Box: 2424, Khartoum-11111, Sudan; ^3^Department of Pharmacology and Toxicology, College of Pharmacy, Umm Al-Qura University, Makkah 21955, Saudi Arabia; ^4^Faculty of Dentistry, Jazan University, P. O. Box 114, Jazan 45142, Saudi Arabia; ^5^Department of Pharmacognosy, College of Pharmacy, Jazan University, P. O. Box 114, Jazan 45142, Saudi Arabia; ^6^Department of Pharmaceutical Chemistry, College of Pharmacy, Jazan University, P. O. Box 114, Jazan 45142, Saudi Arabia; ^7^Department of Microbiology and Immunology, College of Medicine, Alfaisal University, p.O. Box 50927, Takhasusi Road, Riyadh-11533, Saudi Arabia

## Abstract

Many medicinal plants have been utilized for centuries despite the lack of scientific evidence of their therapeutic effects. This study evaluated the phytochemical and dual biological profiling, namely, antibacterial and cytotoxic properties, of three plant species, namely, *Tribulus terrestris* L.*, Typha domingensis* Pers., and *Ricinus communis* L., in order to explore potential relationships (if any) with their ethnopharmacological uses. GC-MS was used to achieve phytochemical screening of two plant extracts (*T. terrestris and T. domingensis*). The primary chemicals detected in varying amounts in both extracts were siloxane derivatives, fatty acid esters, diisooctyl phthalate, phytosterol, and aromatic acid esters. According to the findings, the major component detected in both extracts was 1,2-benzenedicarboxylic acid and diisooctyl ester (antibacterial and antifungal). *T. domingensis* contained a low level of benzoic acid, methyl ester (antibacterial). Both extracts included stigmasterol and sitosterol, as well as six different forms of fatty acid esters. Antimicrobial, antioxidant, anticancer, thyroid inhibitor, and anti-inflammatory properties have all been described. Human breast adenocarcinoma (MCF7), human ovary adenocarcinoma (A2780), and human colon adenocarcinoma (HT29), as well as normal human fetal lung fibroblasts (MRC5), all showed cytotoxic activity. The most potent activity against A2780 cells was seen in *T. terrestris* and *T. domingensis* extracts (IC_50_: 3.69 and 5.87 g/mL, respectively). *R. communis* was more active against MCF7 cells (1.52 *μ*g/mL) followed by A2780 and HT29 cells, respectively. *R. communis* showed a dose-dependent clonogenic effect against MCF7 cells. The antibacterial activity of all three plant extracts was tested against three standard Gram-positive, four standard Gram-negative, and two clinical bacterial strains. Among the three extracts examined, *T. terrestris* was the most effective, followed by *R. communis*, and finally, *T. domingensis* plant extract was effective against various isolated bacteria. This study, interestingly, sheds light on the bioactive components found in plant extracts that can be utilized for cytotoxic and antibacterial purposes.

## 1. Introduction

*Tribulus terrestris* is an annual plant mainly found in subtropical and Mediterranean regions such as China, India, Pakistan, South Africa, Australia, and Europe [1, 2]. The fruits and roots of *T. terrestris* have been used as a folk medicine for many years. The fruits are used in traditional medicine in many countries, including India in Ayurvedic medicines, China in traditional Chinese medicines, and Bulgaria. For the last few years, herbal pharmaceutical preparations containing extracts from *T. terrestris* have been available in several countries due to a wide range of pharmacological activities including antidiabetic, antioxidant, anti-inflammatory, antibacterial, antitumor, improvement in sexual function, and cardio-protective activities [2, 3]. Indeed, several pharmaceutical preparations containing *T. terrestris* extract with mainly steroidal saponins are available worldwide. These preparations are primarily used to manage libido disorders in both men and women and other male sexual disorders; however, data regarding the efficacy of the extracts in such conditions are not concrete.

A wide range of constituents displaying several pharmacological activities with diverse chemical structures has been isolated from *T. terrestris*extracts. These compounds belong to different classes, including steroidal saponins, tannins, flavonoids, alkaloids, glycosides, phytosterols, amino acids, amide derivatives, and proteins. Steroidal saponins and flavonoids are considered the most important constituents with various biological properties. However, the chemical composition of the extract varies based on the extraction and the parts of the plant selected for investigation [2, 3]. *T. terrestris* has been traditionally used in India and China to manage several conditions, mainly for the improvement of sexual functions and prevention and to cure diabetes and cardiovascular disorders.

Although *T. terrestris*has not been extensively studied for detailed anticancer activities, several investigations indicated its potential cytotoxic activity. In a recent study, Patel et al. (2019) reported that methanolic and saponin extracts of *T. terrestris* exhibited cytotoxic activity probably through extrinsic and intrinsic apoptotic pathways [4]. Namely, the seed and leaf extracts induced apoptosis and DNA fragmentation in human breast cancer MCF-7 cells. The extracts also remarkably increased caspase 3/8 activity and upregulated Bax, p53, FADD, and AIF gene expression in MCF-7 cells, in addition to Bcl-2 downregulation. The extracts also reduced the expression of CXCR4 genes, whereas the levels of CCR7 and Bcl-2 genes were decreased in tumorigenic MCF-7 cells [5].

*Typha domingensis* has been traditionally used through topical application for burn and wound healing in Turkish folk medicine [6, 7]. Phytochemical investigation indicated that *T. domingensis* contains many biologically active principles, including polyphenols, hydroxycinnamic acids, flavonoids, and proanthocyanidins. GC-MS analysis of the plant revealed the presence of several long-chain n-alkyl ferulates and n-alkyl-p-coumarates in root and leaf extracts of the plant collected from Florida Everglades. These constituents were not reported in other dominant wetland vegetations [8]. The compounds in *T. domingensis* extracts were identified as essential fatty acids (linoleic acid and *α*-linoleic acid) and phenolic compounds (p-coumaric acid, gallic acid, and caffeic acid) [9]. Akkol et al. reported remarkable wound healing property of 5% ointment formulation prepared from the female inflorescence of *T. domingensis*, when tested in rate mice models. The methanol and aqueous extracts were also influential in wound healing, while the ointment of an extract from male fluorescence was ineffective. They expected that the wound healing property of female inflorescence might be due to the antioxidant property of phenolic contents present in higher concentrations compared to the male inflorescence. In agreement with this study, Chai et al. demonstrated that the extracts of fruits and female inflorescence of *T. domingensis* displayed significant antioxidant activity. The extracts were also effective as iron-chelator and antiglucosidase inhibitors, the latter demonstrating antidiabetic potential of the plant. The crude extract of *T. domingensis* leaf (methanolic and aqueous) demonstrated antimicrobial activities against several Gram-positive and Gram-negative bacteria [10]. Additionally, Majed and Ali demonstrated cytotoxic activity of the aqueous ethanol pollen extract of *T. domingensis* against MCF7 cells (GI_50_ 254 µg/mL), while the same extract showed no effect against MDA-MB-231 cells [11].

*Ricinus communis* L. belongs to the family of Euphorbiaceae and is commonly known as the “castor plant” [12–15]. The plant is quite famous for its typical applications in global traditional and ethnomedicines. The plant has proven its medical potential as the extracts from different parts have recently displayed remarkable bioactivities against several ailments, including paralysis, pain, constipation, diabetes, and wound infections. Also, it is effective as an antioxidant and anti-inflammatory, hepatoprotective, and anticancer agent [15–21]. These medicinal applications are due to the presence of a wide range of phytoconstituents, including terpenoids, flavonoids, alkaloids, anthraquinones, tannins, saponins, polyuronides, glycosides, steroids, and reducing sugars [13, 14, 22]. Several other studies have proven that *R. communis* possess significant antimicrobial activities as it displayed broad-spectrum inhibitory effects against several pathogenic bacterial and fungal strains [18, 23–25]. The fruit extract of *R. communis* demonstrated cytotoxic properties against two breast cancer cell lines MCF-7 and MDA-MB-231 via inhibition of metastatic processes such as adhesion, invasion, cell motility, and reduction of MMP-2/9 expression. The extract was also reported to induce apoptosis by Bax/BCL-2 ratio enhancement, which was also proposed to induce caspases followed by PARP cleavage [15]. Abbas et al. reported that methanolic extracts of leaf, fruit, seed, stem, and root exhibited mild to moderate cytotoxicity against human and bovine RBCs [12]. Our literature survey revealed that *R. communis* is a potential medicinal plant with a rich source of bioactive compounds that may be identified as promising therapeutic agents through advanced techniques.

Although the selected plant species in this study have been screened for various phytochemical and biological activities, these activities need to be linked with ethnopharmacological uses. Therefore, three selected plant species, i.e., *T. terrestris* (whole plant), *T. domingensis* (vegetative part), and *R. communis* (seeds), were selected in our study to evaluate their phytochemical and biological profiles. In this study, GC-MS was used for phytochemical screening of the plant extracts. The selected plants were then evaluated for antibacterial activities against three standard Gram-positive, four standard Gram-negative, and two clinical bacterial strains. Following, the cytotoxicity was investigated using three cancer cell lines in addition to normal fibroblast.

## 2. Materials and Methods

### 2.1. Plant Collection and Extraction

*Tribulus terrestris* L. (whole plant), *Typha domingensis* Pers. (vegetative part ), and *Ricinus communis* L. (seeds) were collected from Western Sudan. Specimens were prepared and subjected to taxonomical identification and authentication at the Medicinal and Aromatic Plants and Traditional Medicine Research Institute, National Center for Research, Khartoum, Sudan.

Extraction was conducted by a slightly modified method previously described by Harborne [26]. Powder of the under shade-dried samples (500 g) was dipped in 2.5 liters of 80% ethanol for 72 h at room temperature with constant shaking. The supernatant was filtered through Whatman filter paper (0.45 µm). This process was repeated twice. The extracts obtained were allowed to dry at room temperature. The extracts were then refrigerated at 4°C in dark bottles until used.

### 2.2. GC-MS Analysis of the Extracts

The extracts were diluted in methanol (1 : 10 v/v) and were analyzed using Thermo Scientific GC-MS equipped with AS 3000 autosampler, trace ultra GC, and ISQ detector. Thermo Scientific TR 5MS column with dimensions of 30 m × 0.25 mm (internal diameter) × 0.25 µm (film thickness) was used to separate the components. At a flow rate of 1.2 mL/min (constant flow mode), helium was used as carrier gas. A volume of 2 µL of sample extracts was injected in split-less mode. The injection port was set at 320°C. The temperature of the oven was initially set at 70°C for 5 minutes, which was subsequently ramped to 205°C at the rate of 5°C/min and held for 5 minutes and then increased to 280°C at the rate of 5°C/min and held for 5 minutes, then to 290°C at the rate of 5°C/min and again held for 5 minutes, and finally to 300°C at rate of 5°C/min and held for 5 minutes. The maximum oven temperature was set at 320°C. The mass spectrometer was operated in an electron ionization (EI) mode within the mass range of 60–900 amu with 0.6 scan times (min). The MS ion source temperature and transfer line temperature were set at 320°C and 350°C, respectively, with an electron multiplier voltage of 1 Kv.

### 2.3. Identification of Phytochemical Constituents

The mass spectra were interpreted using Xcaliber software. The fragmentation patterns in the mass spectra obtained for all constituents were compared with the data stored in the instrument database using the NIST, MAINLIB, and REPLIB built-in libraries. The constituent percentages were measured based on the peak area. The components were identified upon comparison with the structures available in the computer library. The reported biological activities of the constituents listed (Table 1) are taken from Dr. Duke's Phytochemical and Ethnobotanical Database [38].

### 2.4. Cell Culture

Three cancer cell lines, MCF7 (human breast adenocarcinoma), A2780 (human ovary adenocarcinoma), and HT29 (human colon adenocarcinoma), in addition to MRC5 (normal human fetal lung fibroblast), were used in this study. All cells were obtained from the American Type Culture Collection (ATCC). The three cancer cells were cultured in RPMI-1640 (10% FBS) media, while MRC5 was cultured in Eagles minimum essential medium (EMEM, 10% FBS), all at 37°C, 5% CO_2_, and 100% relative humidity.

### 2.5. Cytotoxicity Assay

As previously reported, the cytotoxicity of the three extracts was evaluated by MTT assay [39, 40]. All cancer cell lines and normal fibroblast were separately cultured in 96-well plates (3 × 103/well) and incubated at 37°C overnight. Final extract concentrations were 0, 6.25, 12.5, 25, 50, and 100 *μ*g/mL (DMSO 0.1%; *n* = 3). The 96-well plates were incubated for 72 h, followed by the addition of MTT. Plates were further incubated for 3 hrs, the supernatant was aspirated, and DMSO was added to each well. Absorbance was read on a microplate reader. The optical density of the purple formazan recorded at A550 is proportional to the number of viable cells. IC_50,_ i.e., extract concentration causing 50% inhibition compared to control cell growth (100%), was determined.

### 2.6. Clonogenic Assay

The Clonogenic assay measures tumor cell survival and subsequent proliferative ability following drug exposure [41]. *R. communis* was selected for further clonogenic assay, as it showed the highest selectivity towards the normal cell line MRC5. Exponentially growing MCF7 cells in DMEM (supplemented with 10% FBS and 1% penicillin/streptomycin) were seeded in duplicates at a density of 200 cells/well in a 6-well plate and allowed to attach overnight and then exposed to increasing concentrations of *R. communis* extract (0, 0.5, 1.5, and 2.5 µg/mL) for 72 h. The wells containing media with plant extract were replaced with fresh media without extract. Cells were left to grow at 37°C, 5% CO_2,_ and 100% humidity. Wells were checked daily, and cells forming colonies were roughly counted. After 14 days, plates were rinsed in PBS, fixed with prechilled methanol at room temp for 20 min, then stained with 0.5 methylene blue in 1 : 1 methanol/H_2_O (V/V) for 10 min, and washed in dH_2_O thoroughly and air-dried. Cell colonies were counted macroscopically and reported.

### 2.7. Antimicrobial Assays

#### 2.7.1. Test Microorganisms

The plant extracts were tested against eight reference bacterial strains, *Escherichia coli* (ATCC 25922), *Pseudomonas aeruginosa* (ATCC 27853*), Proteus mirabilis* (ATCC 14153), *Klebsiella pneumonia* (ATCC 700603), *Staphylococcus aureus* (ATCC 20213), *Streptococcus pneumonia* (ATCC 49213), *Staphylococcus epidermidis (*ATCC 12228), *Streptococcus faecalis* (ATCC 29212), and one fungus, *Candida albicans* (ATCC 20231). The tested strains were obtained from the Department of Microbiology, King Fahad Hospital, Jazan, Saudi Arabia.

#### 2.7.2. Preparation of the Test Organisms

A loopful of isolated bacteria colonies were incubated in 4 ml peptone water at 37°C for one hour to adjust the turbidity. The turbidity was adjusted to match the turbidity standard of 0.5 McFarland units [42]. The fungal cultures were maintained on Sabouraud dextrose agar, incubated at 25°C for 4 days. Fungal culture was washed with 100 mL sterile normal saline and refrigerated at 4°C until used.

#### 2.7.3. In Vitro Antimicrobial Testing

The minimum inhibitory concentration (MIC) and minimum bactericidal concentration (MBC) were used to test the antibacterial efficacy of the extract; one ml of the stock bacteria (10^5^–10^6^ CFU per milliliter) was added to 100 ml of Muller Hinton agar at 45°C. Adjusted 20 ml of the previously prepared media were distributed in sterilized Petri dishes. 4 cups of 10 mm diameter were made after settling the medium at room temperature using a sterilized cork borer (No. 4). The holes were filled with 50 µL of the ethanol extracts and kept at room temperature for 1 hr to diffuse and then incubated at 37°C overnight. The diameter of the inhibition zone in Petri dishes was calculated. The same thing was done for antifungal activity; the media was replaced by Sabouraud dextrose agar [43].

#### 2.7.4. Determination of MIC and MBC

The minimum inhibitory concentration (MIC) was detected for all the extracts (*T. terrestris*, *T. domingensis,* and *R. communis*) against the microorganisms using the broth dilution method. Test bacterial cultures (100 *μ*L of bacterial culture containing 10^5^ CFU/mL) were inoculated into tubes containing 25, 12.5, 6.25, 3.12, 1.56, 0.78, and 0.39 mg/L extract concentrations and incubated overnight at 37°C. The values were determined by detecting the inhibition of visible growth in the culture tubes. Similarly, minimum bactericidal concentration (MBC) was detected by subculturing the broth onto freshly prepared Muller Hinton agar medium and at 37°C, overnight. The last concentration of MIC tubes that have not shown any bacterial growth was regarded as MBC [44].

## 3. Results and Discussion

### 3.1. Phytochemical Screening of *T. terrestris* and *T. domingensis* Extracts by GC-MS

**Figure 1:**
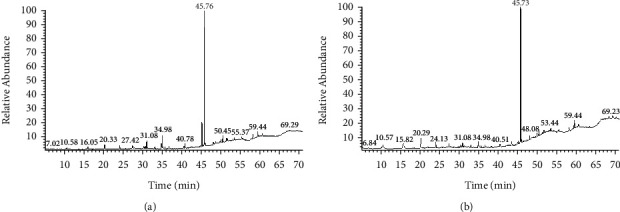
GC-MS chromatogram of (a) *T. terrestris* and (b) *T. domingensis*.

**Figure 2:**
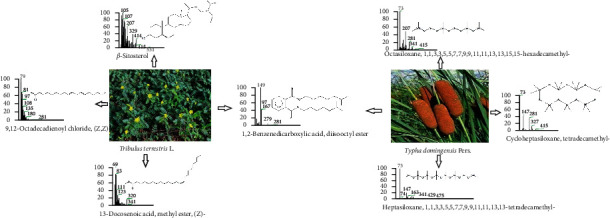
Major phytocompounds present in *Tribulus terrestris* and *T. domingensis*.

Various constituents of *T. terrestris* and *T. domingensis* extracts were efficiently separated and identified using GC-MS (representative chromatograms are shown in Figure 1). As expected, the phytochemical compositions of the extracts varied significantly. Siloxane derivatives, fatty acid esters, diisooctyl phthalate, phytosterol, and aromatic acid esters were the major compounds present in different concentrations in *T. terrestris* and *T. domingensis* plant extracts, as shown in Table 1 and Figure 2. 1,2-Benzenedicarboxylic acid and diisooctyl ester (27.45–34.74%) are present as primary compounds in both the extracts with reported antibacterial and antifungal activity [35]. Benzoic acid and methyl ester (1.82%) were identified in a minor amount in *T. domingensis*, which has been reported to possess antibacterial properties [27]. Different siloxane derivatives were present in both extracts in different concentrations (6.32% in *T. domingensis*). These derivatives are reported to be important in personal care products such as hair and skincare, antiperspirants, and deodorants. Their antibacterial, antifungal [28], insecticidal, antimicrobial [36, 37], and preservative properties [27] have been reported. Six different types of fatty acid esters were identified in both methanol extracts with reported antioxidant, thyroid inhibitor [33], anticancer [34], anti-inflammatory [32], hemolytic, antiandrogenic, lubricant, pesticide, nematicide, hypocholesterolemic [30], and dienophilic [31] activities. Stigmasterol and *β*-sitosterol were only identified phytosterols with a wide range of pharmacological activities such as antimicrobial, anticancer, diuretic, anti-inflammatory, antioxidant, antiasthma, hepatoprotective, and anticancer activities [30, 33].

### 3.2. Cytotoxicity of *T. terrestris*, *T. domingensis,* and *R. communis*

Testing the extracts of *T. terrestris, T. domingensis,* and *R. communis* with MTT assay showed variable activities. *T. terrestris* and *T. domingensis* extracts showed their highest activity against A2780 cells (IC_50_: 3.69 and 5.87 *μ*g/mL, respectively, Table 2). In contrast, *R. communis* was more active compared with the other two extracts, as it showed IC_50_ 1.52 *μ*g/mL against MCF7 cells, followed by 3.04 *μ*g/mL against A2780 and 3.95 *μ*g/mL against HT29 cells, respectively.  Table 3 presents selectivity of three extracts against the normal cells (MRC5), which shows that *T. terrestris* and *T. domingensis* extracts possess highest selectivity (1.79–3.61) to MRC5 compared to A2780 and HT29 cells, while *R. communis* showed significantly high selectivity to MRC5 cells (4.58–11.90) compared to the three cancer cells. *R. communis* extracts have been reported to exhibit anticancer activities against various cancer cell lines in the previous studies as well [15].

**Figure 3:**
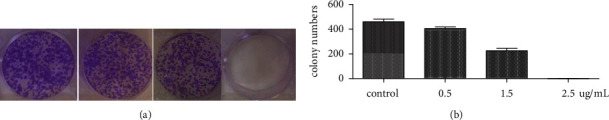
Clonogenic assay; (a) colonies of MCF7 cells treated with *R. communis* for 72 h (from left 0, 0.5, 1, and 2.5 µg/ml; *n* = 3) in 6-well plates followed by 14 days of extract-free incubation; (b) bar graph showing *R. communis* concentrations (*x*-axis) and colony number (*Y*-axis). Results are expressed as cell number ± SD of three independent experiments.

MCF7 cells were the most sensitive to *R. communis,* thus it was chosen to be investigated for its clonogenic activity against MCF7 cells. *R. communis*showed a dose-dependent clonogenic effect against MCF7 cells (Figure 3). Our study stands both as a scientific evidence and support for the previous studies explaining the cytotoxic activity of the three plants. Our results support the previous studies that claimed antitumor activity of *T. terrestris* [2, 3]. In this study, the extract of the whole plant was investigated, while Patel et al., Goranova et al., and Angelova et al. proved both cytotoxic and antiapoptotic effect of its seed and leaf against MCF7 and MCF-10A cells [4, 5, 45]. Previously, Majed and Ali demonstrated cytotoxic activity of the pollen extract of *T. domingensis* against MCF7 cells [11], while the vegetative part of the same plant was tested in this study against the same cell line and showed twelve folds more activity compared to the pollen part. The seeds extract of *R. communis* showed in this study the most significant cytotoxic effect compared to the other two extracts. These results are comparable with previous studies, where *R. communis* fruit, leaf, and volatile extracts showed cytotoxic and apoptotic effects against MCF-7, MDA-MB-231, and SK-MEL-28 cells [15, 46].

### 3.3. Antibacterial Activity

The antibacterial activity of the extracts was determined using the disc diffusion method. The tested extracts showed different activity against different bacteria. *T. terrestris* was the most effective among the three tested extracts, followed by *R. communis*, and finally, *T. domingensis* plant extract was found to be effective against different isolated bacteria; *T. terrestris* was effective against most occurring bacteria, except *K. pneumoniae, S. epidermidis, and S. pneumoniae*. The activity differs in *R. communis* from positive in *Pseudomonas aeruginosa, Staphylococcus aureus, Streptococcus faecalis, and Klebsiella Pneumoniae* and negative in *P. mirabilis*, *E. coli*, *S. epidermidis,* and *S. pneumoniae*. *T. domingensis* showed less activity; it was positive in *Staphylococcus aureus, Streptococcus faecalis,* and *Klebsiella pneumoniae* and negative in the rest of the tested bacteria. The zone of inhibition of the three extracts varied from 10.0 ± 0.01 to 24.5 ± 0.35 in *T. terrestris,* 12.0 ± 0.06 to 18.5 ± 0.24 in *R. communis,* and 8.5 ± 0.35 to 14.0 ± 0.27 in *T. domingensis* (Table 4). *Candida albicans* was found to be insignificant in all of the tested extracts.

MIC and MBC were determined to establish the dose specificity and nature of the activity of the extracts. MIC and MBC values in the extracts varied from one extract to another. In *T. terrestris*, the values ranged from 500 to 6.25 mg/L for MIC and 25 to 12.5 mg/L in the MBC; the values in *R. communis* were 250 to 500 mg/L for MIC and 500–25 mg/L for MBC. The value of MIC was from 250 to 500 mg/L and MBC was from 0 to 500 mg/L in *T. domingensis*.

The antibacterial results of the extracted plants were comparable to those of the reference medication gentamicin, which exhibited substantial results against the bacteria tested, showing that the antimicrobial results were reasonable.

Our finding proved the antimicrobials efficacy of the tested extracts among different tested bacteria. Our results of *T. terrestris* are not far from what was reported by Abdulqawi and Quadri (2021); their results illustrated the antibacterial efficiency of methanolic extract of *T. terrestris* fruits against *E. coli, P. aeruginosa, P. mirabilis, K. pneumoniae,* and methicillin-resistant strain of *S. aureus* [47]. In another in vitro study, the plant's aqueous extract and benzoxacin fraction were effective against about 50% of *H. pylori* strains [48]. *T. terrestris* extracts in different solvents were tested on *Bacillus cereus*, *Staphylococcus aureus,* and *Escherichia coli,* and the methanolic extract was found to be most effective against all bacterial strains. The other extracts which showed comparatively lesser potency were in ethanol, petroleum ether, and chloroform [49]. Soleimanpour et al. (2015) reported the antibacterial activity of ethanolic extract of *T. terrestris* against *Actinomyces viscosus, Staphylococcus aureus, Streptococcus mutans, Streptococcus sanguinis, Enterococcus faecalis,* and *Escherichia coli* [50]. The same study reported that the complex extract of *T. terrestris, Capsella bursa-pastoris,* and *Glycyrrhiza glabra* exhibited superior and synergistic effects compared to individual extracts. Various other studies revealed antibacterial activity of *T. terrestris* extracts against a number of Gram-positive and Gram-negative bacterial strains [51–54]. All previous studies and our findings proved the antimicrobials efficacy of *T. terrestris*.

The present study supports the previous findings that showed that *R. communis* possesses significant antimicrobial activities against several tested bacteria and this resembles what was found by [18, 23–25].

The antimicrobial results of *T. domingensis* were near to what was found by Al-Kalifawi et al. (2017), who demonstrated antimicrobial activities against several Gram-positive and Gram-negative bacteria, including *Streptococcus species, Staphylococcus aureus, Bacillus cereus, Enterobacter cloacae, Klebsiella pneumonia, Pseudomonas aeruginosa,* and *Escherichia coli* [10].

## 4. Conclusions

The ethanolic extracts of *Tribulus terrestris* L. and *Ricinus communis* L. yielded fifteen bioactive phytochemicals, which were reported to possess antibacterial, antifungal, antioxidant, anticancer, thyroid inhibitor, and anti-inflammatory properties.

The extracts showed variable cytotoxic properties against cancer cell lines. The most active extracts were *T. terrestris* and *T. domingensis* against human ovarian cancer cells, whereas *R. communis* was more active against human breast adenocarcinoma cells. The clonogenic impact of *R. communis* on human breast cancer cells was dose-dependent. The most efficient antibacterial agent was *T. terrestris*, while *T. domingensis* was the least effective against several isolated bacteria. The antibacterial findings of the extracted plants were comparable to those of the reference medication gentamicin, which exhibited substantial effects against the tested bacteria, implying that the antimicrobial results were reasonable.

The findings of this research justify the ethnopharmacological uses as antimicrobial in wound healing. However, the other ethnopharmacological application is still to be verified scientifically through proper pharmacological screenings.

## Figures and Tables

**Table 1:** Chemical composition of the aerial parts of *T. terrestris* and *T. domingensis* extracts by GC-MS.

S. No.	RT	Name of the compound	Molecular formula	Molecular weight	Area% *Tribulus terrestris* L.	Area% *Typha domingensis* Pers.	Nature of compound	Structure	Pharmacological activity
1	10.57	Benzoic acid, methyl ester	C_8_H_8_O_2_	136	—	1.82	Aromatic acid ester	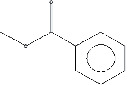	Antibacterial [27]
2	16.05	Cyclohexasiloxane, Dodecamethyl-(D6)	C_12_H_36_O_6_ Si_6_	444	1.31	—	Siloxane deriv.	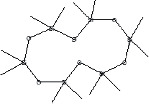	Used in personal care products such as hair/skin care products, antiperspirants, and deodorants; antibacterial and antifungal [28]
3	20.33	Cycloheptasiloxane, tetradecamethyl-	C_14_H_42_O_7_ Si_7_	518	1.67	3.19	Siloxane deriv.	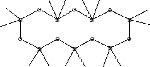	Preservative [27]
4	24.15	Cyclooctasiloxane, hexadecamethyl-	C_16_H_48_O_8_ Si_8_	592	1.01	—	Siloxane deriv.	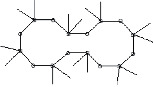	Antimicrobial [27]
5	27.42	Heptasiloxane, 1,1,3,3,5,5,7,7,9,9,11,11,13,13-tetradecamethyl-	C_14_H_44_O_6_ Si_7_	504	1.04	3.10	Siloxane deriv.	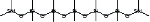	Insecticidal activity [29]
6	31.08	Hexadecanoic acid, methyl ester	C_17_H_34_O_2_	270	1.67	—	Fatty acid ester	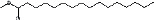	Hemolytic, antiandrogenic, lubricant, pesticide, nematicide, antioxidant, hypocholesterolemic [30]
7	34.79	Methyl 9-cis,11-trans-octadecadienoate	C_19_H_34_O_2_	294	1.34	—	Fatty acid ester	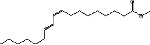	Dienophilic activity [31]
8	34.94	9,12,15-Octadecatrienoic acid, methyl ester, (Z,Z,Z)-	C_19_H_32_O_2_	292	—	1.55	Fatty acid ester	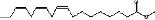	Anti-inflammatory [32]
9	34.96	9,12-Octadecadienoyl chloride, (Z,Z)-	C_18_H_31_ClO	298	5.97	—	Alkyl chloride	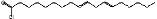	Antioxidant, anticancer, thyroid inhibitor [33]
10	40.78	Methyl 9,10-methylene-octadecanoate	C_20_H_38_O_2_	310	1.29	—	Fatty acid ester	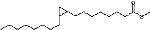	No activity
11	45.15	13-Docosenoic acid, methyl ester, (Z)-	C_23_H_44_O_2_	352	5.98	—	Fatty acid ester	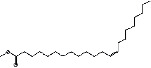	Anticancer [34]
12	45.76	1,2-Benzenedicarboxylic acid, diisooctyl ester (diisooctyl phthalate)	C_24_H_38_O_4_	390	34.74	27.45	Phthalic acid diester	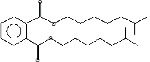	Antibacterial, antifungal [35]
13	58.19	Stigmasterol	C_29_H_48_O	412	1.63	—	Phytosterol	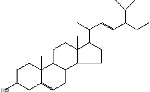	Antimicrobial, anticancer, diuretic, anti-inflammatory, antioxidant [33]
14	59.44	*β*-Sitosterol	C_29_H_50_O	414	1.80	2.40	Phytosterol	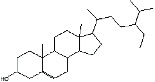	Antiasthma, hepatoprotective, diuretic, antimicrobial, anti-inflammatory, anticancer [30]
15	60.54	Octasiloxane, 1,1,3,3,5,5,7,7,9,9,11,11,13,13,15,15-hexadecamethyl-	C_16_H_50_O_7_ Si_8_	578	1.43	16.51	Siloxane deriv.	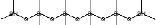	Antimicrobial [36], insecticidal activity [37]
Total identified phytocompounds		71.57	56.02						

^*∗*^ Reported activity obtained from Dr. Duke's phytochemical and ethnobotanical database [38].

**Table 2:** Cytotoxic activity of *T. terrestris, T. domingensis,* and *R. communis *extracts against three cancer cell lines and one normal fibroblast (MTT 72 h, IC_50_ ± sd *μ*g/mL).

Extract	IC_50_			
	MCF7	A2780	HT29	MRC5
*Tribulus terrestris* L.	14.83 ± 0.79	3.69 ± 0.36	32.84 ± 3.88	13.31 ± 0.98
*Typha domingensis* Pers.	21.02 ± 1.44	5.87 ± 0.87	7.31 ± 1.25	13.11 ± 1.08
*Ricinus communis* L.	1.52 ± 0.67	3.04 ± 0.49	3.95 ± 0.90	18.09 ± 0.57

**Table 3:** Selectivity of the three extracts against MRC5 normal cells.

Extract	IC_50_	SI^*∗*^		
	MRC5	MCF7	A2780	HT29
*Tribulus terrestris* L.	13.31 ± 0.98	0.90	3.61	0.41
*Typha domingensis* Pers.	13.11 ± 1.08	0.62	2.23	1.79
*Ricinus communis* L.	18.09 ± 0.57	11.90	5.95	4.58

SI: selectivity index = IC_50_ value of extract against normal MRC5 cells/IC_50_ value of the same extract against either MCF7, A2780, or HT29 cells.

**Table 4:** Diameter zone of inhibition, minimum inhibitory concentration (MIC), and minimum bactericidal concentration (MBC) of *T. terrestris, T. domingensis,* and *R. communis* extract*s*.

Organism	Plant extract									Gentamicin (+ve control)
	*Tribulus terrestris* L.			*Typha domingensis* Pers.			*Ricinus communis* L.			
	Zone of inhibition (mm, mean ± SEM)	MIC (mg/L)	MBC (mg/L)	Zone of inhibition (mm, mean ± SEM)	MIC	MBC	Zone of inhibition (mm, mean ± SEM)	MIC (mg/L)	MBC (mg/L)	
*Proteus mirabilis*	11.5 ± 0.22	12.5	25	—	—	—	—	—	—	22.0 ± 0.05
*Pseudomonas aeruginosa*	24.5 ± 0.35	12.5	25	—	—	—	18.5 ± 0.24	12.5	25	20.0 ± 0.08
*Staphylococcus aureus*	11.5 ± 0.35	6.25	12.5	9.5 ± 0.36	500	—	16.5 ± 0.23	12.5	25	21.0 ± 0.02
*Streptococcus faecalis*	14.5 ± 0.34	12.5	25	14.0 ± 0.27	250	500	13.5±0.23	250	500	23.0 ± 0.05
*K. Pneumoniae*	—	—	—	8.5 ± 0.35	500	—	12.0 ± 0.06	250	500	18.5±0.23
*E.coli*	10.0 ± 0.01	500	—	—	—	—	—	—	—	17.0±0.24
*S. epidermidis*	—	—	—	—	—	—	—	—	—	17.0 ± 0.09
*S. pneumoniae*	—	—	—	—	—	—	—	—	—	25.0 ± 0.08
*Candida albicans*	—	—	—	—	—	—	—	—	—	—

## Data Availability

All the data related to the study are available with the corresponding author and can be provided on request.
